# Robot-Assisted Therapy in Guillain–Barrè Syndrome: Systematic Review of Primary Evidence and Study Protocol for a Randomized Clinical Trial

**DOI:** 10.3390/jcm13237153

**Published:** 2024-11-26

**Authors:** Alex Martino Cinnera, Martina D’Arienzo, Diego Piatti, Laura Casagrande Conti, Pietro Deledda, Alberto Tenore, Stefano Paolucci, Maria Grazia Grasso

**Affiliations:** 1 Scientific Institute for Research, Hospitalisation and Health Care IRCCS Santa Lucia Foundation, 00179 Rome, Italy; a.martino@hsantalucia.it (A.M.C.); m.darienzo@hsantalucia.it (M.D.); laura.casagrandeconti@gmail.com (L.C.C.); s.paolucci@hsantalucia.it (S.P.); mg.grasso@hsantalucia.it (M.G.G.); 2 School of Physiotherapy, Faculty of Medicine and Surgery, University of Rome Tor Vergata, 00133 Rome, Italy; deleddapietro03@gmail.com (P.D.); alberto.tenore2203@gmail.com (A.T.)

**Keywords:** robotic rehabilitation, exoskeleton device, polyradiculoneuropathy, chronic inflammatory demyelinating polyradiculoneuropathy, polyradiculopathy

## Abstract

**Background:** Guillain–Barré syndrome (GBS) is an immune-mediated polyradiculoneuropathy that represents a leading cause of motor impairment. Robot-assisted therapy (RAT) has been widely applied in various neurological conditions. However, the use of RAT in GBS remains underexplored. This systematic review (SR) aims to evaluate the preliminary evidence regarding the efficacy of RAT in terms of motor recovery in people with GBS (pwGBSs). Secondly, the study protocol for a randomized RCT is reported. **Methods:** A comprehensive SR was conducted on PubMed, Scopus, EMBASE, Cochrane Library, and Epistemikos. Risk of bias was assessed using the National Institute of Health (NIH) study quality assessment. The SR’s protocol was recorded in the PROSPERO database. **Results:** Out of 116 articles found, four studies published in the past four years met the inclusion criteria. These studies investigated the effects of RAT on lower limbs (three studies) and upper limbs (one study) in four pwGBSs. The results showed improvements in motor function and patient engagement, but it is impossible to generalize the findings. **Conclusions:** Our SRs supports the rationale for an RCT to assess the efficacy of RAT in pwGBSs. We present the protocol for a double-blind RCT to evaluate the effects of RAT on upper limb motor function in pwGBSs.

## 1. Introduction

Guillain–Barré Syndrome (GBS) is a leading cause of acute flaccid paralysis, presenting with limb weakness and hyporeflexia or areflexia [1,2]. GBS is an immune-mediated polyradiculoneuropathy, with around 100,000 new cases reported worldwide annually [3]. The mean age of onset is 40, affecting more males than females, across all ages and demographics [4]. GBS incidence varies seasonally and is linked to infections, peaking in winter in Western countries and in summer in East Asia and Latin America, where Campylobacter jejuni infections are associated with acute motor axonal neuropathy (AMAN) [5]. Rising Campylobacter infections globally may contribute to maintaining or increasing GBS incidence. Sensory symptoms, such as paraesthesia or numbness, typically begin distally in a symmetrical pattern [2]. Several GBS variants are recognized. Acute inflammatory demyelinating polyneuropathy (AIDP), the most common form, was first identified over a century ago. In AIDP, the immune system attacks peripheral nerve myelin with secondary axonal involvement [4]. AMAN is a purely motor variant characterized by primary axonal degeneration, rapid progression, preserved or exaggerated tendon reflexes, and rarely, autonomic dysfunction [5]. Acute motor and sensory axonal neuropathy, a recently described subtype, involves acute sensorimotor symptoms, loss of deep tendon reflexes, and distal weakness [6]. Miller Fisher syndrome, a rare variant first described in the 1950s, is characterized by ataxia, areflexia, and ophthalmoplegia. Incomplete forms include acute ophthalmoplegia, ataxic neuropathy, ptosis, and mydriasis. Ptosis occurs in 60% of cases, while facial nerve palsy affects 30–50% of patients. Sensory deficits and weakness occur in 20–50% and 20–25% of cases, respectively [7]. A pharyngeal–cervical–brachial variant, affecting 3% of GBS cases, is marked by rapid oropharyngeal and cervicobrachial weakness with hyporeflexia or areflexia in the upper limbs and is associated with axonal neuropathy rather than demyelination [8]. Many rehabilitative approaches have been studied to improve motor outcomes in people with GBS (pwGBSs). In the current rehabilitation context, robot-assisted therapy (RAT) is widely used to improve motor outcomes in people with stroke [9,10,11], in spinal cord injuries [12], or multiple sclerosis [13], as well as other neurological diseases [14]. However, the evidence about the effectiveness of RAT on motor outcomes in pwGBSs remains under recorded. As with other neurological conditions that benefit from RAT, this innovative approach may also offer a promising additional treatment for pwGBSs. Furthermore, given the rapid advancement and continuous integration of robotic devices into rehabilitation settings, extending their use to pwGBSs could optimize patient recovery and contribute to cost-effectiveness. The aim of this systematic review was to evaluate the preliminary evidence about the efficacy of RAT on motor outcomes in pwGBSs. Furthermore, the results of our SR are used to support the rationale for a randomized double-blind clinical trial. In the study protocol, we describe procedures to test the efficacy of RAT on upper limb (UL) motor function, ability in activities of daily living, quality of life, and manual strength and dexterity in pwGBSs.

## 2. Methods

A systematic review to investigate the effectiveness of RAT on motor outcomes in pwGBSs was conducted from inception to 1 April 2024. The protocol was recorded in the PROSPERO database on 7 October 2024. The research was performed on five databases: PubMed, Scopus, EMBASE, Cochrane Library, and Epistemikos, using terms to identify the intervention (i.e., robotics) and the disease (i.e., Guillain–Barré syndrome) (the complete search strategy is available in the Supplementary Materials). The research is reported following the Preferred Reporting Items for Systematic Reviews and Meta-Analyses (PRISMA) guidelines and the research questions were formulated according to the PICO strategy (population: pwGBS; intervention: RAT; control: conventional therapy; outcome: motor outcome). The PRISMA flowchart is available in the Supplementary Materials. Inclusion criteria were as follows: (I) evaluation of RAT effects on motor outcome; (II) study performed on adults with GBS; (III) full text available; (IV) English language. Exclusion criteria were as follows: (I) people with other diseases; (II) conference papers or study protocols; (III) non-English language; (IV) studies performed on children. All studies were uploaded to an online dataset (RYYAN) [15]. Screening and data extraction were performed by two blinded reviewers (P.D. and A.T.) and in case of disagreement, a third reviewer (M.D.A.) was involved in the judgment.

### Risk of Bias Assessment

The risks of bias in the four studies were analyzed with the NIH “Quality Assessment Tool for Before–After (Pre–Post) Studies with No Control Group” [16]. Although our systematic review included some randomized controlled trials (RCTs), these RCTs did not have Guillain–Barré syndrome (GBS) subjects in their control groups. For this reason, to ensure an equitable and robust evaluation of risk of bias across all studies included in our review, we chose the NIH Quality Assessment Tool for Before–After (Pre–Post) Studies with No Control Group, organized according to question numbers from the tool for quality assessment of controlled intervention studies: (I) study question; (II) eligibility criteria and study population; (III) study participants representative of clinical populations of interest; (IV) all eligible participants enrolled; (V) sample size; (VI) intervention clearly described; (VII) outcome measures clearly described, valid, and reliable; (VIII) blinding of outcome assessors; (IX) follow-up rate; (X) statistical analysis; (XI) multiple outcome measures; (XII) group-level interventions and individual-level outcome efforts. Results were interpreted as good, fair, of poor. Two blinded reviewers assessed the risk of bias and in case of disagreement, a third reviewer was consulted.

## 3. Results

A total of 116 articles were found; after screening titles and abstracts, seven articles were included in the full-text analysis. Two blinded reviewers conducted full-text analysis. In case of disagreement, consensus was achieved following review by a third author. Finally, four articles met the inclusion criteria [17,18,19,20] and were included in the synthesis.

### 3.1. Participant

The first study [20] involved four neurological patients (two stroke, one Guillain–Barrè, one spinal cord injury), all requiring gait rehabilitation. The second study [19] included 11 adult inpatients with a mean age of 64.4 years (±11.2). The diagnoses were distributed as follows: eight patients had suffered a stroke, two had spinal cord injuries, and one had Guillain–Barré syndrome. A reference group consisting of another set of 11 inpatients with a mean age of 64.3 years primarily had diagnoses of stroke and spinal cord injury. Two case reports were included in the review, about a patient of 78 years old [17] and a patient of 75 years old [18]. Additional details on the studies are given in Table 1.

### 3.2. Interventions

The first study [20] investigated the effectiveness of integrating a socially assistive robot (SAR) for gait rehabilitation in patients with neurological disorders, including one patient with GBS. This SAR was a type of robotic system designed to engage with patients through social interactions to provide assistance, support, or companionship. It provides social interaction and emotional support during sessions, contributing to improved patient engagement. The researchers used a treadmill-based walking machine (Lokomat^®^, Hocoma, Volketswil, Switzerland) consisting of a harness to carry patients in an upright position and robotic arms attached to the patient’s legs, allowing physiological and symmetrical reciprocal movement on a treadmill [21]. The research involved four neurological patients and utilized a repeated measurement design to compare therapy sessions with and without the SAR. The two case reports that we included in the review were also focused on gait rehabilitation. Yabuki et al. [17] employed an ABAB design, alternating between a conventional gait program (Phase A) and the use of a gait trainer (Phase B) providing dynamic support and feedback on movements. This robotic gait trainer was used to assist patients during walking and functional activities such as stair climbing. The patients utilized additional assistive devices such as a dynamic plastic ankle–foot orthosis and a walker as needed. In contrast, Chen et al. described two robotic-assisted gait training (RAGT) sessions with Lokomat^®^. The study by De Crignis and colleagues [19] explored the application of a robotic arm in one patient with GBS. This device was designed to support rehabilitative exercise for the upper limbs, enabling patients to perform specific movements even in the presence of muscle impairment. The primary focus was on how this robotic intervention could aid recovery by assisting patients in performing therapeutic exercises that they may have found difficult due to muscle weakness and coordination challenges associated with GBS. Finally, Chen et al. [18] studied RAT in walking rehabilitation, finding it to be a feasible and safe intervention for enhancing ambulation and functional abilities in geriatric pwGBSs; the case study was conducted across two Lokomat sessions.

### 3.3. Control

In the first study [20], the control condition involved standard gait rehabilitation without assistance from the SAR. The effectiveness of the SAR was evaluated by comparing outcomes between rehabilitation sessions assisted by the SAR and those conducted under the control conditions. In the second study, [19] participants in the control group received treatment using commercially available robotic therapy devices for the UL, such as ArmeoPower^®^ or ArmeoSpring^®^ (both Hocoma). It should be noted that feasibility studies could include patients with various types of neurological disorders, not always affected by GBS, making it impossible to establish a reliable control group. Usability and comfort were assessed through standardized questionnaires, including the Quebec User Evaluation of Satisfaction with Assistive Technology (QUEST) and the Raw Task Load Index (RTLX).

### 3.4. Outcomes

The first study’s outcomes [20] showed improvements in thoracic and cervical posture, with notable gains in both areas when using the socially assistive robot (SAR). Patient engagement levels and overall satisfaction with the therapy process were also evaluated. The second study’s outcomes [19] included usability and satisfaction ratings from both patients and therapists, perceptions of workload using the Raw Task Load Index (RTLX), and visual perceptions of the augmented reality (AR) gaming scenario. Key parameters measured during the sessions included muscle activation, range of motion, and patient feedback regarding perceived exertion and comfort levels. The studies by Yabuki [17] and Chen [18] utilized a range of outcome measures to assess the effectiveness of their respective gait rehabilitation interventions. Yabuki et al. measured key gait parameters, including comfortable walking speed, stride length, and cadence at the start of each training phase, alongside secondary outcomes such as GBS disability score, MRC score for muscle strength, the 6 min walk test for walking endurance, motor-FIM for independence in activities of daily living (ADLs), and the Overall Neuropathy Limitations Scale for neuropathy-related limitations. Chen et al. assessed functional outcomes at discharge using the Barthel Index for ADL independence, the clinical frailty scale, and the Instrumental Activities of Daily Living (IADL) Scale. In general, the studies showed heterogeneity in the outcome measures used and in the number of sessions conducted (Table 1); however, they present promising results for the use of RAT in this patient population.

### 3.5. Risk of Bias Results

**Figure 1:**
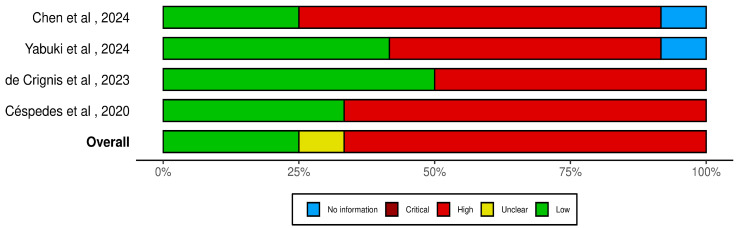
Risk of bias in the included studies [17,18,19,20] evaluated with the NIH “Quality Assessment Tool for Before–After (Pre–Post) Studies with No Control Group”.

The four studies reported in the lower panel (Figure 1) showed high risk of bias in relation to Questions 3 and 5, which pertain to study participants and sample size. The study participants of all studies were not representative of clinical populations of interest; two studies were case reports [17,18] with only one pwGBS described, while the samples in the two RCTs [19,20] included different neurological conditions and were not sufficiently large to provide confidence in the results for pwGBSs. Also, standards relating to Question 8 and 9 were judged as “poor” for all studies, due to lack of blinding of assessors and follow-up. Any statistical methods were described in the case reports, while Cèspedes and de Crignis reported a statistical analysis that examined changes in outcome measures before and after the intervention in the experimental group. Regarding Questions 2 and 4, only the two RCTs [19,20] provided prespecified and clearly described inclusion criteria for their study populations, but they did not mention the exclusion criteria. In summary, the included studies presented an overall high risk of bias and were deemed inadequate to provide replicable results for pwGBSs, due to limitations in both study design and study population.

## 4. Study Protocol

As indicated by the literature review, a randomized controlled study with an adequate sample size is mandatory to investigate the effects of RAT for upper limb (UL) recovery in pwGBSs. A parallel two-arm randomized clinical trial has been planned according to the CONSORT statement [22].

### 4.1. Aim of the Study

The aim of this study is to evaluate the effects of RAT on motor recovery in patients with GBS. The primary endpoint is the assessment of UL motor function and fine motor skills after treatment with and without RAT. Secondary endpoints include improvements in overall quality of life and the patient’s general health status.

### 4.2. Trials Design

We will conduct a double-blind randomized controlled clinical trial with two parallel arms.

### 4.3. Eligibility Criteria

Inclusion criteria are as follows: (i) diagnosis of GBS; (ii) UL motor impairment (0–4 on the Medical Research Council scale); (iii) sub-acute phase (<180 days); (iv) age between 18 and 80 years; (v) patients must be able to maintain a sitting position. Exclusion criteria are as follows: (i) concomitant neurological, orthopedic, metabolic, and oncological diseases; (ii) cognitive impairment (mini mental state examination < 24); (iii) visual deficit; (iv) hearing disorders.

### 4.4. Sample Size

The sample size was calculated according to data reported for the control group undergoing conventional rehabilitation in a previous study [23], which reported a median Barthel Index of 75 at discharge with an interquartile range of 36 (from which a standard deviation of 26 was estimated). Assuming a 25% greater improvement in the experimental group, with a significance level (alpha) set at 5% and a test power of 80%, the required sample size was calculated to be 31 patients per group, for a total of 62 patients.

### 4.5. Randomization and Blinding

This study is a double-blind, randomized, controlled clinical trial (RCT). A computer-generated randomization list will be used to produce block randomization according to block size. The randomized list will be securely stored by a researcher in a password-protected cloud. It will not be made available to the researcher responsible for the assessments. Also, patients will remain blinded to their group assignment.

### 4.6. Interventions

Patients will be randomized in two different groups:

Experimental Group (RAT): Patients will undergo 20 sessions of 45 min each with an exoskeleton for upper limb rehabilitation. The assistance provided by the device will be adjusted based on the maximum force (as a percentage of the upper limb’s weight) the robot needs to exert to assist the patient’s movements. Each session will include exercises designed to improve the range of motion (ROM) of the shoulder, elbow, and wrist and to enhance hand coordination. The training parameters including level of difficulty, duration, and visual stimuli will be adjusted based on the patient’s residual abilities. The selected exercises may involve movements of a single joint along one axis, combined movements of a single joint around 2 or 3 axes, selective exercises for opening and closing the hand, or multi-joint exercises. All exercises will be realized with audiovisual feedback.

Control Group (Ctrl): Patients will undergo 20 sessions of 45 min of neurorehabilitation intervention with a sham treatment under the guidance of the experimental therapist. This will involve passive mobilization using the exoskeleton for the upper limb, without any audiovisual feedback. They will not realize any active movement during the training.

### 4.7. Outcome Measurements

Assessments will be performed before treatment at T0 (day one) and at the end of treatment, then at T1 (30 days). Two follow-ups will be performed, one at T2 (60 days) and another follow-up at T3 (90 days). The assessments will be performed considering the following outcome measures. The Fugl-Meyer assessment scale for upper extremities (FMAUE) will be used to assess the change in the motor and sensory functions of the UL. The FMA includes five domains: motor functioning, sensitivity, coordination, range of motion, and pain [24]. Scoring ranges from 0 to 66 points; a higher score corresponds to a better function of the UL. The modified Barthel Index (mBI) will be used to assess the change in the autonomy of patients during their activities of daily living (ADLs). This scale has a score ranging from 0 to 100 points; a higher score corresponds to a better performance in ADLs [25]; The 36-Item Short-Form Health Survey (SF-36) will be used to assess the change in health-related quality of life. The SF-36 is organized into eight different domains that are divided into physical components (physical functioning, limitations due to physical problems, bodily pain, and general perceptions of health status) and mental components (social functioning, general mental health, limitations due to emotional problems, and vitality). The eight domain scores are added together and the result is linearly transformed on a scale from 0 to 100, where 0 equals negative health and 100 equals positive health [26]. ABILHAND will be used to assess manual dexterity. ABILHAND is a questionnaire-based assessment tool that measures the difficulty a patient perceives in using their hands to perform manual operations and activities in daily living; it assesses upper limb functionality. It has nonlinear logit values ranging from 1.72 to −2.18 [27]. The Medical Research Council scale (MRC) will be used to assess muscle strength. Scores range from 0 to 5, where 0 equals no muscle recruitment and 5 normal muscle recruitment [28]. The nine-hole peg test (NHPG) will be used to assess fine manual dexterity. The NHPT consists of a square board with nine pegs. At one end of the board are holes to insert the pegs, and at the other end is a shallow round plate to store the extracted pegs. The NHPT is administered by asking the patient to take the pegs from a container, one by one, and insert them into the holes on the board, as quickly as possible [29]. Grip strength will be assessed using a manual digital dynamometer. The maximum strength of the whole hand will be recorded three times without feedback (with the patient sitting with his back to the monitor). The measurement will be performed using the PABLO suite in Tyromotion^®^ (Hocoma) (CE ID-BD/RDM: 1634215).

### 4.8. Data Collection and Management

All collected data will be stored electronically through an interface compliant with European data protection regulations (GDPR No. 679/2016) and Italian regulations (D.L. 101/2018), as well as any subsequent amendments and regulations issued by the Data Protection Authority. Personal and contact information will be pseudonymized and password-protected. This information will be recorded in a separate dataset.

### 4.9. Statistical Analysis Plan (SAP)

Demographic characteristics and clinical variables will be investigated with normality tests (Shapiro–Wilk) in order to determine the distribution. All normally distributed data (*p* > 0.05) will be analyzed by repeated-measure analysis of variance (rmANOVA) 2 × 4 (two groups × four times). The intragroup analysis will be performed as a function of the TIME variable, the between-groups analysis will be performed by exploring the interaction of TIME*GROUP. All statistically significant results (*p* < 0.05) will be corrected with a Bonferroni post hoc test. Non-parametric data will be investigated by non-parametric ANOVA (Friedman test). All statistically significant results will be analyzed via a Wilcoxon *t*-test on the differences in scores between the evaluation intervals (ΔTIME).

### 4.10. Ethical Considerations and Informed Consent

This study was approved by the Ethics Committee of Lazio Area 5, on 24 July 2024 (No. 110/SL/24). The current version of the protocol is version 4.0. A written informed consent form will be signed by each participant. The study “Upper Limb Robot-Assisted Therapy in Patients with Guillain–Barré Syndrome (RAUL Project)” has also been recorded in Clinicaltrial.gov and was released in 1 October 2024 (NCT06620198).

### 4.11. Safety and Monitoring

Studies that have used RAT have not reported significant adverse events (AEs) [30]. However, in one study, cases of discomfort and the presence of vesicles at the level of the fingers were reported [31]. Any adverse effects will be communicated to the PI.

### 4.12. Timeline

**Figure 2:**
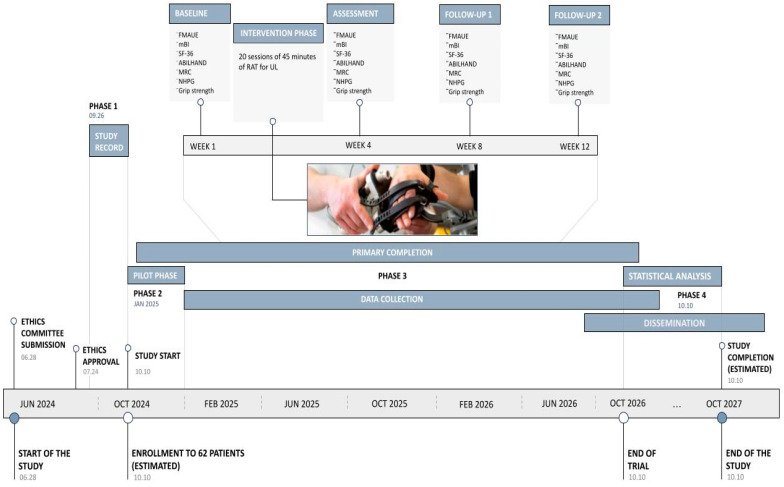
Roadmap of RAUL Project. Ph. credit: IRCCS Santa Lucia Foundation Hospital.

The clinical trial is scheduled to begin in 2024, with patient enrollment expected to be completed over a two-year period. Following this, a period will be required to conduct statistical analysis and disseminate the results. The study is anticipated to be completed by October 2027 (Figure 2).

## 5. Discussion

The aims of the present study were to identify evidence about the effectiveness of RAT in pwGBSs and to present a study protocol to improve knowledge in the field of GBS rehabilitation. A limited number of studies have been conducted to investigate the effectiveness of RAT for motor recovery in pwGBSs. We found four studies involving only four pwGBSs. No AEs were recorded, confirming the safety of the use of robotic devices in neurological rehabilitation. Regarding the effects, although the study resulted appear promising with regard to improving motor function, the limited evidence does not permit the generalization of results, and a well-designed RCT is needed. Moreover, the limited number of GBS patients involved in these studies and the sometimes low-quality methodology are significant limitations in interpreting any results. Nevertheless, the rationale behind the use of RAT in rehabilitation suggests promising prospects for its application by clinicians to support the recovery of pwGBSs. The potential benefits of using RAT for GBS are considerable, especially given its alignment with neuroplasticity principles that could facilitate motor recovery. Indeed, RAT delivers task-specific exercises that embody core neuroplasticity principles such as repetition, intensity, and targeted practice, which can promote neural reorganization and consequently, motor function. This approach could also enhance rehabilitation by making it more comfortable and increasing patient engagement. RAT has been studied in other neurological diseases, demonstrating its efficacy in motor recovery [9,10,11,12,13,14]. It provides a complementary strategy for use with conventional rehabilitation. For example, robot-assisted gait therapy can help in the initial rehabilitation steps after stroke, compensating for the patient’s postural weakness by using lumbar straps attached to the device and motorized joint-control assistance, while conventional therapy can be focused on trunk stability or sit-to-stand training [32]. These complementary approaches can be task-oriented simultaneously, while RAT can be useful in maintaining the high intensity of tasks. Task-oriented activity and high-intensity rehabilitation are key factors in enhancing the use-dependent plasticity of the sensorimotor cortex [33]. Probably, the differences in recovery mechanisms between the central nervous system, spinal cord, and peripheral nervous system have contributed to the limited focus on RAT in pwGBSs. Additionally, the low incidence of GBS may have led to reduced research attention. Future studies are essential in order to better understand the potential of RAT in this population. The variability of outcome measures used to date also makes it necessary to consider new trials to understand which are responsive to rehabilitation treatment, in order to allow their use in clinical settings. The two studies considered were characterized by a methodology that did not allow collection of high-quality evidence. In light of this, it will be essential that future randomized controlled trials conducted on this topic are well designed and include randomized control groups.

### Limitations

The study protocol provides for the enrollment of 62 patients. Since GBS is a rare disease, this number, although seemingly small, may still be difficult to achieve. For this reason, one year after the start of the study, an evaluation will be conducted regarding the sample size. Then, the study will either be extended or proposed for transformation into a multicenter study.

## 6. Conclusions

We hypothesize that RAT can be useful in the management of upper limb deficits caused by peripheral neuropathy. In this context, we believe that a new clinical trial demonstrating the efficacy and safety of RAT in pwGBSs is necessary. From a rehabilitative point of view, if our results confirm the effectiveness of RAT in pwGBSs’ rehabilitation, we could improve rehabilitation programs by integrating this novel approach, which would complement conventional therapies and allow treatment strategies tailored to the specific needs of each individual.

## Figures and Tables

**Table 1:** Summary of studies using RAT in pwGBSs.

Author (Year) Country [Ref.]	Study Design	Sample N (pwGBSs), Gender, Mean Age ± SD, (Range)	Procedure N Duration (Session × Week)	Outcome	Results	Conclusions
Céspedes (2020) Colombia [20]	No randomized controlled study (feasibility study)	RAT + NAO robot group 4 (1) 3 M NR (20–60) Ctrl group 4 (1) 3 M NR (20–60)	RAT + NAO robot NR (1 × 1) RAT NR (1 × 1). Each patient performed two RAT sessions on Lokomat: one control and one assisted by the social NAO robot, carried out on different days of the week. (1) Therapy time should be the same during the sessions; (2) each patient should perform a unique session per condition; and (3) all Lokomat features must be the same across therapy sessions.	Cardiovascular parameters (Zephyr HxM BT), spinal posture parameters (cervical and thoracic IMU), and Borg scale.	The results of the study showed a positive and well-received effect of the robot regarding postural behavior in the cervical and thoracic area, companionship, and social interaction. In the robot-assisted scenario, patients relied more on the feedback and paid more attention.	High motivation was provided by the robot to perform the therapy with good posture. Therapists and patients agreed that the robot had been helpful in the session.
de Crignis (2023) Germany [19]	No randomized controlled study (feasibility study)	RAT group 11 (1) 9 M 64.4 ± 11.2 (47–85) Ctrl group 11 (0) 9 M 64.3 ± 9.1 (49–79)	RAT group NR (4–5 × NR). Patients performed four to five sessions of upper limb rehabilitation with a RobExReha device. Ctrl group, 4–90 min NR. Trained with ArmeoPower or ArmeoSpring, both with an average of 15 (±25 min) therapy sessions completed.	Patients: QUEST, RTLX, HoloLens, and pAR questionnaire. Therapists: completed subscale of QUEST, SUS and UEQ-short to evaluate perceptions of the device’s usability.	The patients’ usability ratings were significantly higher in the Reference Group for two items of the QUEST: reliability and ease of use. Workload (RTLX) ratings did not differ significantly between the groups. Nearly all patients using the RobExReha system perceived the gaming scenario in AR as functioning adequately despite eight patients having impairments in stereoscopic vision. The therapists valued the system’s approach as interesting and inventive.	Therapy with the RobExReha system was safe and feasible for patients and therapists, with no serious adverse events being reported. This structured approach allowed researchers to systematically evaluate the role of social robots in enhancing rehabilitation outcomes for individuals with mobility impairments, providing valuable insights into future applications of robotics in therapy.
Yabuki (2024) Japan [17]	Case report	GBS (1) 78	ABAB design Phase A 10–40 min (6 × 10). Conventional gait program included level walking and treadmill gait training. Phase B 20–30 min (5 × 6). Gait trainer HWA-01 included HGT during physical therapy (walk and climb stairs).	CWS, stride length, and cadence at the start of training in all phases. GBS disability score, MRC sum, 6MWT, motor-FIM, and ONLS.	The study reported significant improvements (*p* < 0.05) in the patient’s functional mobility following the use of the exoskeleton. Specific metrics of mobility enhancement were documented, indicating that robotic assistance could play a vital role in rehabilitation for pwGBSs.	Exoskeleton robots could be beneficial for patients with GBS experiencing ongoing gait disturbances. This case report highlighted the potential for such technologies to augment traditional rehabilitation methods, suggesting further research into larger cohorts and long-term effects.
Chen (2023) Taiwan [18]	Case report	GBS (1) 75	RAT with Lokomat add-on rehabilitation program 54 min (Two sessions) First session included 80% reduction in body weight, treadmill speed of 1.2 km/h, distance of 421 m. The second included 70% reduction in body weight, treadmill speed of 1.3 km/h, distance of 623 m.	BI, CFS, IADLs, CGA, SPPB, SACR-F, GDS, CAM, MMSE.	The findings revealed that robot-assisted gait training led to significant improvements in multiple areas. Participants showed increased lower limb muscle strength following the intervention. Additionally, there was a notable enhancement in functional independence, as reflected in the improved BI scores, indicating better performance in daily activities. Furthermore, the CFS results demonstrated a reduction in frailty levels, suggesting an overall improvement in the patients’ functional status.	RAGT is a feasible and safe intervention for improving ambulation and functional capabilities in geriatric pwGBSs. The robotic approach seems to have many potential benefits in rehabilitation programs for this patient population.

Key: Ctrl, control group; RAT, robot-assisted therapy; IMU, inertial measurement unit; QUEST, Quebec User Evaluation of Satisfaction with Assistive Technology; RTLX, row task load index; pAR, “presence in augmented reality”; SUS, system usability scale; UEQ-short, user experience questionnaire; HWA, hip-wearable exoskeleton; CWS, comfortable walking speed; motor-FIM, motor-functional independence measure; 6MWT, six-meter walking test; ONLS, overall neuropathy limitations scale; BI, Barthel Index; CFS, clinical frailty scale; IADLS, Instrumental Activities of Daily Living Scale; CGA, comprehensive geriatric assessment; SPPB, short physical performance battery; SARC-F, Strength, Assistance in Walking, Rise from a Chair, Climb Stairs, and Falls questionnaire; GDS, geriatric depression scale; CAM, confusion assessment method; MMSE, mini mental state examination.

## Data Availability

No new data were created for the present work.

## Supplementary Materials

The following supporting information can be downloaded at https://www.mdpi.com/article/10.3390/jcm13237153/s1, Search strategy [34].
