# Supplementary figures and images for: Robot-Assisted Therapy in Guillain–Barrè Syndrome: Systematic Review of Primary Evidence and Study Protocol for a Randomized Clinical Trial
Source: J Clin Med. 2024 Nov 26;13(23):7153. doi: 10.3390/jcm13237153 (PMC11641865; doi:10.3390/jcm13237153)

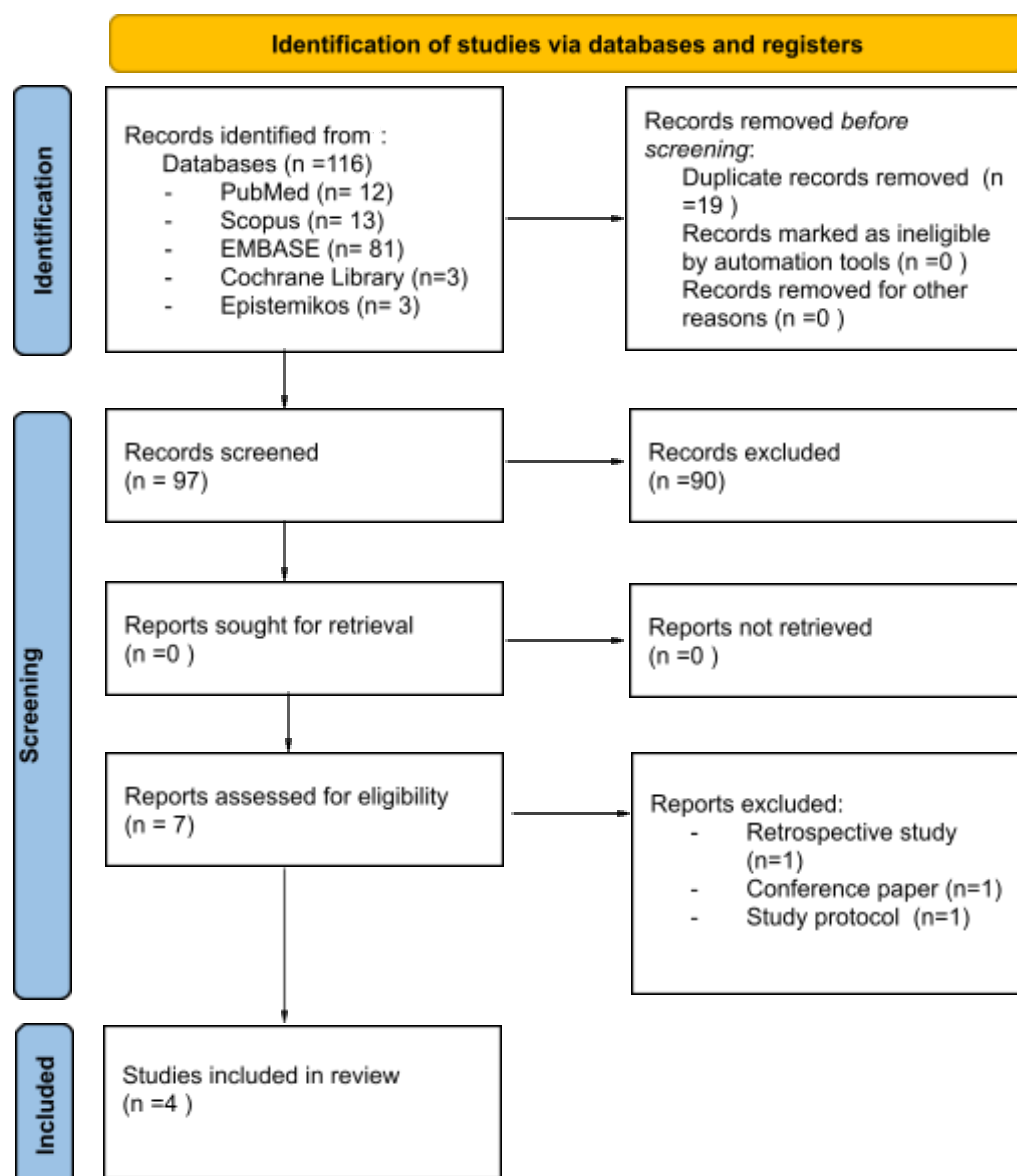

Supplement: Supplementary file 1 [file jcm-13-07153-s001.zip › jcm-3287219-supplementary.pdf]
